# Adaptive Neuro-fuzzy Inference System Trained for Sizing Semi-elliptical Notches Scanned by Eddy Currents

**DOI:** 10.1007/s10921-019-0648-8

**Published:** 2019-12-19

**Authors:** Ehsan Mohseni, Martin Viens, Wen-Fang Xie

**Affiliations:** 1 Department of Electronics and Electrical Engineering, Center for Ultrasonic Engineering (CUE), Technology and Innovation Centre, University of Strathclyde, 99 George Street, Glasgow, G1 1RD UK; 2 Département de génie mécanique, L’École de technologie supérieure, 1100 Rue Notre-Dame O, Montréal, QC H3C 1 K3 Canada; 3 Department of Mechanical & Industrial Engineering, Concordia University, 1455 De Maisonneuve Blvd. W., Montréal, QC H3G 1M8 Canada

**Keywords:** Eddy current testing, Finite element modeling, Split-D reflection differential probe, Tilt and lift-off study, Adaptive neuro-fuzzy inference system

## Abstract

The present study explores the capability of COMSOL Multiphysics, as a finite element modelling (FEM) tool, to model the interaction between a split-D differential surface eddy current (ECT) probe and semi-elliptical surface electrical discharge machined (EDM) notches. The effect of the small probe’s lift-off and tilt on its signal is investigated through modelling and subsequently, the simulation outcomes are validated using the probe’s impedance measurements. In the next stage, an adaptive neuro-fuzzy inference system (ANFIS) is designed to take the signal features as inputs and consequently, provide the length of the scanned notch as the system’s output. The system is trained by extracted features of thirty model-generated signals obtained from scanning of the same number of semi-elliptical notches by means of the split-D probe. The trained ANFIS is tested afterwards using the measured signals of 3 calibration EDM notches together with 5 model-based ones. A very low average estimation error is observed with regard to the length estimation of the test notches and the accuracy of the length estimation is found to be quite reasonable.

## Introduction

Depending on the configuration of eddy current testing (ECT) probes, diverse analytical and semi-analytical models have been in use to study the interaction of these probes with surface and near surface flaws. The growing demand for new applications of ECT adds to the complexity of ECT probes and hence, leads to adoption of numerical approaches for their analysis more than before. From such probe categories, reflection differential split-D probe as a conventional ECT surface probe has served long in nuclear and aerospace industries owing to the spatial configuration of its differentially connected receiver coils. It has a very small footprint, high signal to noise ratio for tiny surface breakings and less sensitivity to gradual variations of both conductivity and permeability as compared to absolute configurations [1, 2]. The impedance evaluation for this probe as it scans defective components requires finding a solution to the forward problem developed based on Maxwell’s equations. Most of these problems are tackled using semi-analytical approaches and deriving integral equations for electromagnetic fields using Green’s dyadic functions [3–5]. Followed by the these approaches, different studies investigated the performance of such probes through the developed volume integral code and some promising outcomes were presented using the method [4, 6]. However, the availability of diverse numerical tools nowadays allows one to treat the problems such as the interaction between complex probe geometries and defects more realistically without the need of evaluating the complicated Green’s function. Finite element modelling (FEM), as a well-known numerical method, is also very distinguished for its flexibility in processing complex geometries and has grown in popularity in ECT analysis in recent years. Unfortunately, the potential of the FEM in modelling of Split-D probes with non-axisymmetric geometries has not fully been explored yet. Accordingly, in the present study, FEM is exclusively used to compute the impedance of a split-D probe as it scans over 30 semi-elliptical electrical discharge machined (EDM) notches with different dimensions in order to form a size dependent signal archive. It is noted that semi-elliptical EDM notches are used to generate impedance trajectories since they can fairly be representative of surface fatigue cracks in terms of the shape [7].

Employing various quantitative non-destructive testing techniques for crack sizing has become very important since the reliance of new structural life estimation approaches and maintenance decision-makings on accurate sizing has grown in various industries [8, 9]. Reliable defect characterization and sizing through inversion of ECT signals can help one optimize the maintenance intervals which could result in the prevention of safety issues as well as the reduction in inspection costs. Therefore, due to their high importance, the inversion of ECT signals has been the concern of many studies in past. Most of the related publications are developed based on the Green’s function with solution to ECT forward problem in form of volume integrals and the inversion is commonly carried out through minimizing a cost function representing the difference between the predictions made by the forward problem and the measurements of the probe [10, 11]. ECT Inversion based on these approaches requires low computational resources and they are fairly fast and accurate. However, some preliminary assumptions regarding the medium and flaw are required. Although, FEM is not as fast as the other techniques and thus, can not be used directly in inversion, one can effectively use FEM-generated impedance trajectories of surface notches in conjunction with artificial intelligence (AI) to produce a sizing scheme. Furthermore, the primary task to adopt such a scheme is ensuring the reliability of FEM predictions for ECT signals.

AI techniques such as fuzzy logic, neural networks, and genetic algorithms are developed based on the inspirations from biological and behavioural nature of the human brain, and they have been used in many real world applications. Integrating two or three of these techniques can be advantageous in designing intelligent soft computing systems [12]. Soft computing has been in use in the field of flaw characterization and classification using ECT signals for a while [13–15]. Among all these approaches, neural networks (NN) and fuzzy logic (FL) are frequently used together either in series or as an integration to form hybrid systems in which the reasoning and inference power of the FL can be complemented by adaptive learning nature of NN.

In this paper, a hybrid system integrating NN and FL has been proposed for the notch length prediction. In FL, the values are transformed to membership degrees of linguistic information through fuzzy set theory by applying linguistic labels to which membership functions are assigned. Subsequently, a fuzzy inference system (FIS) composed of if–then rules is defined based on human perception and serves as the reasoning engine. Finally, the system outputs are defuzzified to quantitative values. Although the FIS is structured based on the human expertise, it does not adapt to changing environment. In order to optimize a designed FIS through training with sets of input/output data, an adaptive neuro-fuzzy inference system (ANFIS) can be of benefit. The training procedure for these systems requires several training data sets comprising of both known flaw dimensions and features of their corresponding signal. Fabricating such samples containing flaws with given dimensions is really costly and time consuming whereas modelling them by FEM allows one to generate as many signals as required to train ANFIS to cover a desired dimensional range for a certain flaw type. Therefore, in the present study ECT signals of the probe scanning over thirty notches with diverse lengths are obtained through FEM simulations in COMSOL Multiphysics®. Afterwards, their signals are post-processed to extract the features which are fed as inputs to an ANFIS for training purposes. The initial Sugeno type FIS has the notch length as output. The trained system is tested subsequently using the measured signals of three calibration notches as well as the model-generated signals of 5 arbitrary semi-elliptical EDM notches.

In industries, the demand for automated inspections is growing every day. Automation can eliminate the errors introduced by human factor as well as reducing the cost and time of inspections. However, there are also a few drawbacks linked to automated scans. For instance in the case of ECT automation, the probe manipulation is of high importance since even a very small tool alignment/positioning error affects the ECT signal significantly. In ECT, such errors appear in form of probe’s tilt and lift-off. Presence of any of these two can change the nature of the recorded signals of scanned flaws, which in turn introduces errors in crack characterizations. Therefore, the impact of small variations of the probe’s lift-off and tilt on ECT signal is investigated through FEM as it scans notches. Subsequently, the extent of the error introduced into sizing caused by these small probe lift-offs and tilt angles is examined through feeding their signal features into the trained FIS.

This study is organized in the following order. In section two, the experimental setup and FEM preparations are explained. Initially, the credibility of FEM for predicting the probe’s signal as the tilt and lift-off vary is assessed by comparing their simulation results to the impedance measurements. Afterwards, the effect of probe’s tilt angle and lift-off variations on ECT signals of three calibration notches is investigated through simulations. In addition, a series of simulations concerning thirty semi-elliptical notches possessing different lengths and depths is performed and consequently, a size dependent table of the notch signals is formed [16]. In section three, each of the signals is analyzed by means of a MATLAB script in order to extract their features such as the peak to peak amplitude, the maximum width and the shape type. Besides, an initial fuzzy inference system is generated having the signal features as inputs and notch length as its output. The set of inputs and output related to each notch is prepared in form of vectors and used for training of ANFIS based on the hybrid method composed of back propagation and least squares optimization methods. Subsequently, the trained FIS is tested by the signals obtained from the 3 calibration notches. Additional tests are carried out using the model-based simulated signals of 5 EDM notches randomly sized within the length size interval used in the training. At last, the signals acquired from the three calibration notches as the probe’s tilt angle and lift-off varies are analyzed. Following that, the extracted features of these signals are fed into the trained ANFIS to explore the level of error introduced in notch length estimation as a result of probe’s tilt and lift-off variation.

## Experiments and Modelling

**Fig. 1:**
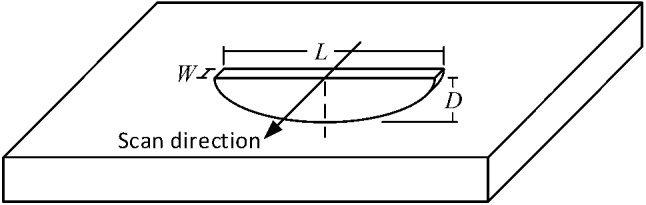
Geometrical features of a semi-elliptical notch

**Fig. 2:**
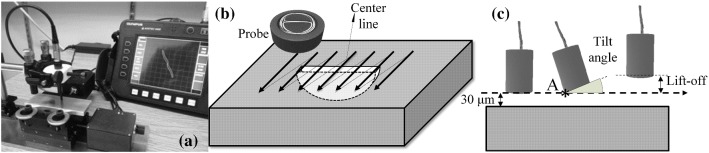
**a** Setup for measuring the probe’s impedance as it scans the notches, **b** direction of the raster scan relative to the notch length, and **c** representation of the lift-off distance and tilt angle applied to the probe

Three semi-elliptical EDM notches in an aluminium 7075-T6 sample, as listed along with their dimensions in Table 1, are used for experimental measurements. The dimensional features of these notches are shown in Fig. 1. The thickness of the aluminium sample is 6 mm and it is extremely thick as compared to the penetration depth of eddy currents at selected test frequency of 500 kHz. A reflection differential split-D surface pencil probe working in frequency range of 500 kHz to 3 MHz together with a Nortec 500S ECT unit are used for scanning of the notches and recording the signals. The probe is fixed within an alignment device. One can set a tilt angle for the probe using this device. The sample is mounted on an X–Y micrometric table which can be programmed to follow a scan trajectory. The experimental set up is demonstrated in Fig. 2a. An initial lift-off of 30 µm is introduced to the probe and maintained during all the experimental and modelling scans. The probe is oriented in a way so that the flat surface of D-cores, cutting a cylinder in half, becomes parallel to the notch side wall. In addition, the perpendicularity of the probe to the sample’s surface is ensured by checking the symmetry of the 8-shaped signal obtained from the scan of a through width calibration surface notch. The notch wall is perpendicular to the surface of the sample, and it is expected the notch produces a symmetrical 8-shaped signal. Afterwards, raster scans are conducted on each of the 3 notches in the way shown in Fig. 2b. The scans are performed with an index of 0.1 mm when the probe is tilted 0°, 2° and 4°. Figure 2c shows the tilt angle introduced to the probe while an initial lift-off of 30 µm is in place. In this figure, the tilt axis, which is perpendicular to the scan direction, passes through point A and is parallel to the surface of the sample. After recording the signals for tilt angle variations, notch signals are also acquired at probe’s lift-offs of 30 µm, 100 µm and 140 µm with no tilt. The impedance data recorded via a data acquisition card possessing a high sampling rate relative to the scan speed. The scan data is then analyzed using a MATLAB script to indicate the scan line being the closest to the line which cuts the notch length in half (i.e. notch center line). Clearly, the accuracy of finding the scan line passing over the notch center line depends on the scan index, symmetry of the notch relative to the plane cutting it in half along its length, the probes internal imperfections and, the surface waviness and non-parallelism.

**Table 1:** Geometry of semi-elliptical EDM notches

Notch	Length, L (mm)	Depth, D (mm)	Opening, W (mm)
A	2.84	1.11	0.1
B	1.62	0.63	0.1
C	0.81	0.31	0.1

**Fig. 3:**
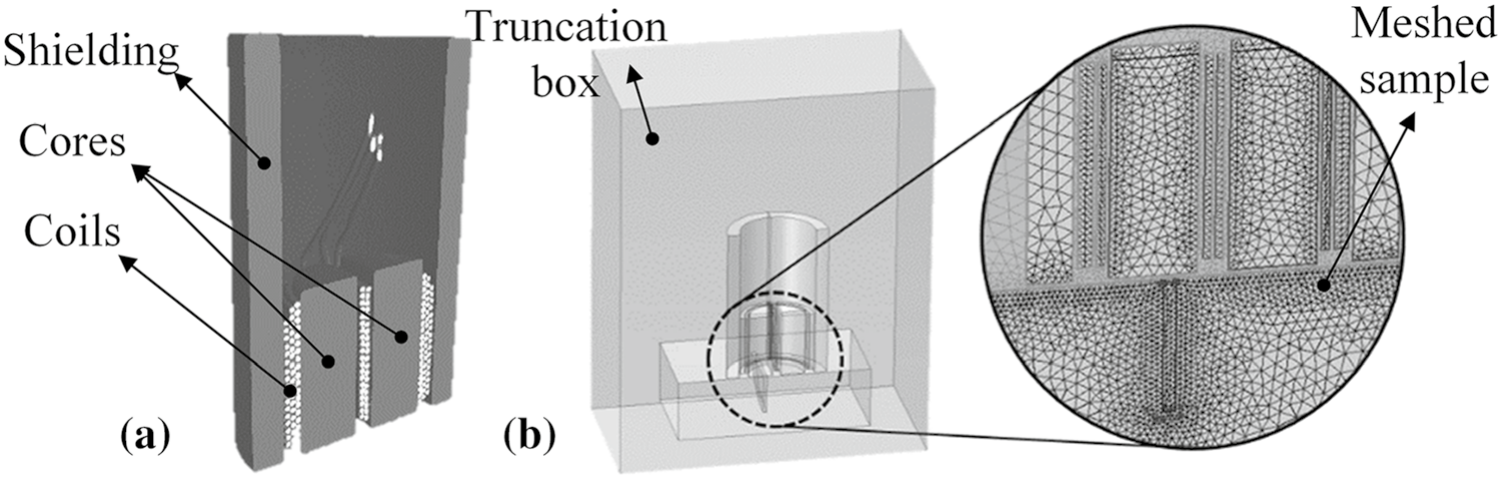
**a** Cut in half 3D reconstruction of X-Ray tomography images of the split-D probe, **b** the probe and sample assembly model prepared within COMSOL Multiphysics^®^ showing a small meshed region in close proximity to the notch geometry

For the purpose of modelling, the three dimensional (3D) CAD model of the probe is generated and imported into COMSOL Multiphysics®. The model is cut in half along its symmetry plane which is parallel to the scan direction. Dimensions of the probe geometry, which are presented in detail previously [16], are extracted from X-ray tomography reconstruction. The X-ray scans are carried out using a Nikon XTH 225 micro focus tomography unit and the reconstructed model is post processed via VG studio MAX. A schematic of the cut in half 3D rendering of the stack of the probe’s X-ray images is provided in Fig. 3a. Besides, the half-scaled 3D CAD model including the assembly of the probe and the sample prepared in COMSOL Multiphysics® along with a small region of the mesh which is applied to the model are illustrated in Fig. 3b. As shown in the figure, in order to shorten the simulation’s run time and save resources, a box encapsulating the probe and sample is defined to truncate the solution domain.

Followed by the assembly preparation, material properties for each domain are defined. Where the relative permeability of 2500 is assigned to the probe cores and the sample’s electrical conductivity is considered as 17.8 MS/m. Subsequently, a pre-defined magnetic field physics equation formulated as Eq. 1 within AC/DC module of COMSOL Multiphysics® is employed and the magnetic insulation boundaries are placed on the faces of the truncation box.

1 $$ ({\nabla \times (\nabla } \times \varvec{A}))/\mu_{\text{0}} \mu_{\text{r}} + (\text{j}\sigma \omega - \omega^{\text{2}} \varepsilon_{\text{0}} \varepsilon_{\text{r}} )\varvec{A} = \varvec{J}_{e} ,\text{j} = \sqrt { - 1} $$

In Eq. 1, $\varvec{A}$ is the magnetic vector potential, $\varvec{J}_{e}$ is the externally applied current density, $\varepsilon_{0}$ and $\mu_{0}$ are the electrical permittivity and magnetic permeability of the free space, respectively. $\varepsilon_{\text{r}}$ and $\mu_{\text{r}}$ stand for the relative electrical permittivity and magnetic permeability of each domain of the model, respectively. In addition, $\sigma$ and $\omega$ are the electrical conductivity and angular frequency. In order to mesh the problem geometry, second order tetrahedral elements are used for each domain. The mesh global size within each domain is adjusted considering the constraints dictated by geometry of each domain and it is locally refined for regions where a high concentration of eddy currents is expected. Therefore, the element size is set to be 0.08 mm on the surface of the sample. The size value is almost half the standard penetration depth of eddy currents ($\delta$) in aluminium at the frequency of 500 kHz. Following that, a small element growth rate of 1.1 is assigned to the sample volume. In this manner, the size of elements across the first few mesh layers under the surface remains below 0.09 mm. Similarly, as depicted in Fig. 3b, a locally finer mesh is generated along the notch walls where eddy current density is expected to be high due to the flow perturbation caused by the notch. Afterwards, an iterative solver with frequency domain analysis step is used to run simulations. In each simulation, the notch is scanned by the probe having 0.1 mm displacement increments. This is defined as a parametric sweep step within the solver. Noteworthy, in all simulations presented here except those of the probe tilt study, the probe and the notch are both perpendicular to the surface of the sample. Therefore, there is a plane-symmetry for the probe’s receiver coils and this plane is also parallel to the notch length during the scans. Accordingly, the notch signals are entirely symmetric relative to the impedance plane’s origin and because of that, only a half of the scan can be simulated for each notch. In this manner, the scan starts where the probe is centered with the notch and it continues until the notch is completely passed by the probe. Consequently, the probe finishes its scan at the position of 1.3 mm away from the notch center where it is located over the undefective region of the sample. However, in tilt study the probe’s full scanning path, which is twice 1.3 mm, is used since the signal is not symmetric anymore. Differential impedance of the probe at each scan position is calculated using Eq. 2. It is assumed that the induction current flowing through receiver coils is significantly smaller in magnitude as compared to the driver coil’s current. Therefore, the equation formulates the impedance changes as the voltage difference of the receiver coils divided by the driver’s current.

2 $$ \Delta \varvec{Z} = ({\text{V}}_{{\text{R2}}} - {\text{V}}_{{\text{R1}}} )/{\text{I}}_{\text{D}} $$

where $\varvec{V}_{{\text{R1}}}$ and $\varvec{V}_{{\text{R2}}}$ are the voltages induced across the receiver coils 1 and 2, respectively and, $\varvec{I}_{\text{D}}$ stands for the current flowing in the driver coil.

Simulations presented in the subsequent sections are categorized in the following order:

1. The scan of calibration notches as the probe has lift-offs of 60 μm, 80 μm, 100 μm, 120 μm, and 140 μm are simulated. The model-based signals obtained at lift-offs of 100 μm and 140 μm are compared with measurements to validate the study.
2. The scan of the calibration notches as the probe’s tilt angle varies from 1° to 4° with steps of 1° are simulated. The model-based signals obtained at tilt angles of 2° and 4° are compared with measurements to validate the study.
3. The signal of thirty notches possessing different sizes, as presented in Table 2, are simulated to establish a size dependent signal archive for ANFIS training.

**Table 2:** Dimensions of semi-elliptical EDM notches used in the simulations

Notch no.	D*	L**	Notch no.	D	L	Notch no.	D	L	Notch no.	D	L	Notch no.	D	L
1	0.3	0.30	7	0.3	0.45	13	0.3	0.60	19	0.3	0.75	25	0.3	0.90
2	0.5	0.50	8	0.5	0.75	14	0.5	1.00	20	0.5	1.25	26	0.5	1.50
3	0.7	0.70	9	0.7	1.05	15	0.7	1.40	21	0.7	1.75	27	0.7	2.10
4	0.9	0.90	10	0.9	1.35	16	0.9	1.80	22	0.9	2.25	28	0.9	2.70
5	1.1	1.10	11	1.1	1.65	17	1.1	2.20	23	1.1	2.75	29	1.1	3.30
6	1.3	1.30	12	1.3	1.95	18	1.3	2.60	24	1.3	3.25	30	1.3	3.90

*Depth in millimetres

**Length in millimetres

All the semi-elliptical notches used in these simulations have 0.09 mm of opening.

### Lift-Off and Tilt Studies

**Fig. 4:**
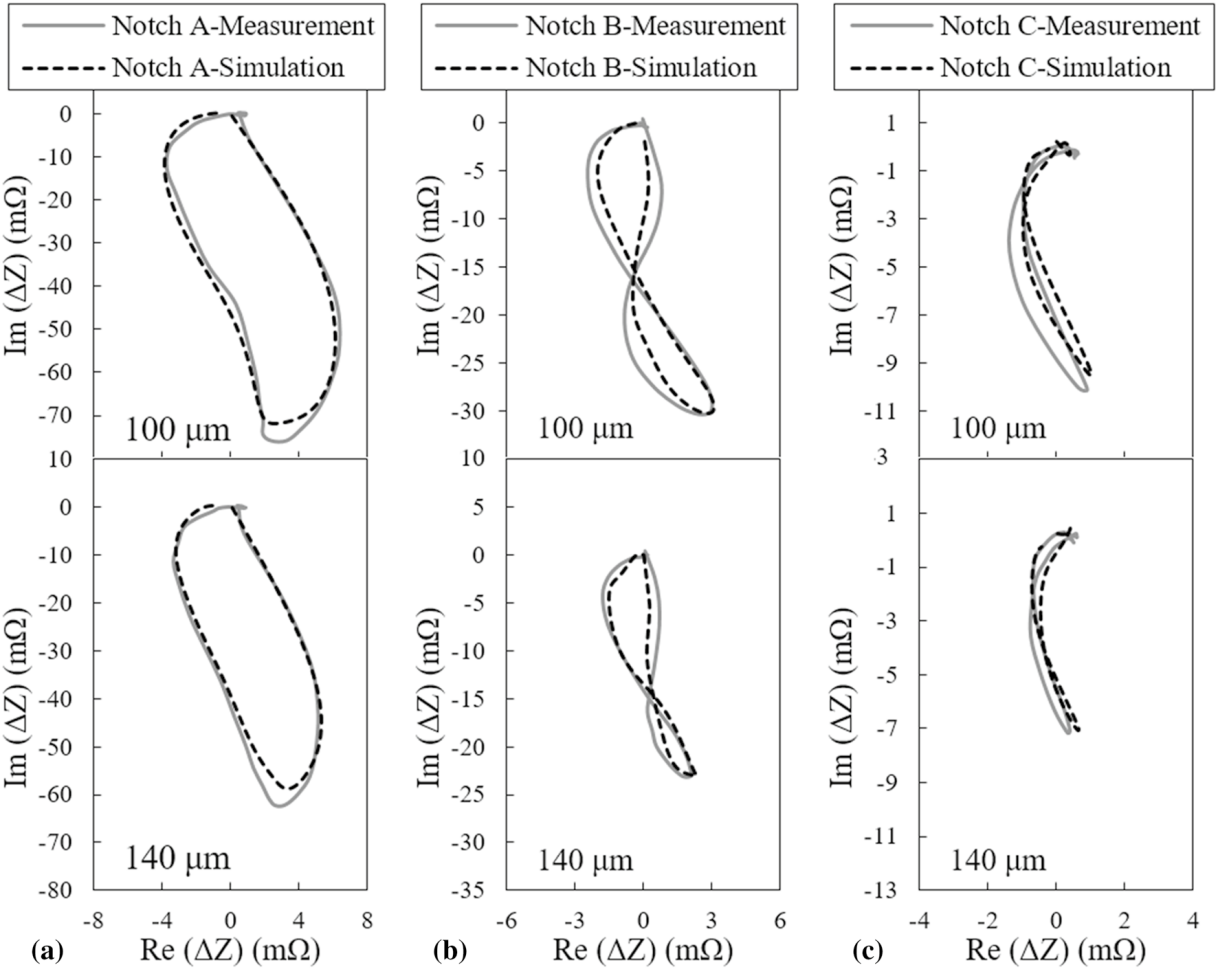
Comparison between the measured and simulated signals of **a** notch A, **b** notch B, and **c** notch C at two different lift-offs of 100 and 140 µm

The results of FEM simulations for the probe’s lift-offs of 100 μm and 140 μm when it scans the three calibration notches of Table 1 are plotted along with the impedance measurements carried out under the same conditions in Fig. 4.

The nature of the small discrepancies that could be observed between the simulations and measurements results in Fig. 4 vary depending on the notch dimensions. These differences can be categorized into three classes:

- The difference in maximum signal amplitude that can be mainly observed for the signals of notch A as demonstrated in Fig. 4a. This type of discrepancy is believed to be caused by one point calibration which is used for adjusting the initial lift-off of the probe. Depending on the initial spot chosen for lift-off calibration, the actual probe’s lift-off might slightly alter as the probe scans locations apart from the calibration point due to the possible non-parallelism of the sample’s top and bottom surfaces as well as surface waviness. It shall be noted that all three notches are located at a certain distance from each other on the same sample and changing the scan spot can affect the lift-off of the probe.
- The shape discrepancy between the simulated and measured signals is mostly present for the smaller notches B and C as can be noticed in Fig. 4b and c. This difference type is most likely associated to the deviations of the actual notch geometry from the ideal one used in simulations. This kind of difference is more visible for notches B and C as compared to notch A since their signature on impedance plane is smaller and more complex. In addition, the EDM manufacturing method that is used to carve these notches reaches its limitations for the small sized B and C notches and hence, the likelihood of deviation from the nominal geometry becomes higher.
- The impedance plane’s origin for simulated signal slightly shifts relative to the one from measurement for notch C according to Fig. 4c. This difference mostly comes to sight for the smallest notch for which a high device gain is used during the measurements. This effect is connected to the nulling procedure used in simulations where the impedance of the probe scanning the defective parts of the sample is subtracted from the probe’s impedance when it is located on undefective parts. Depending on the mesh inhomogeneity in sample, the impedance may not be correctly nulled. Besides, the measured signal is not essentially passing the origin of impedance plane as there is a slight unbalance between the shape of the probe’s cores [16]. The difference might as well be present for the larger notches however, owing to their higher signal amplitudes, the origin shift is negligible relative to the size of the impedance trajectory.

**Fig. 5:**
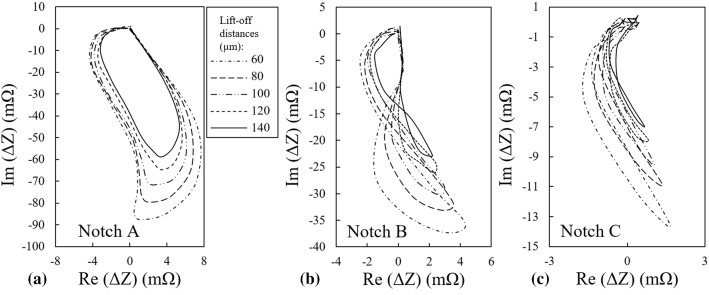
Effect of lift-off variations on the ECT signal of **a** notch A, **b** notch B, and **c** notch C

Despite of these discrepancies, the predictions made by simulations are reasonably good and the model can be used further to study the signal variations as the lift-off changes. Consequently, for each notch, three more scans at probe’s lift-offs of 60 μm, 80 μm and 120 μm are simulated and resulting signals along with the previous ones as the lift-off varies are presented in Fig. 5.

**Fig. 6:**
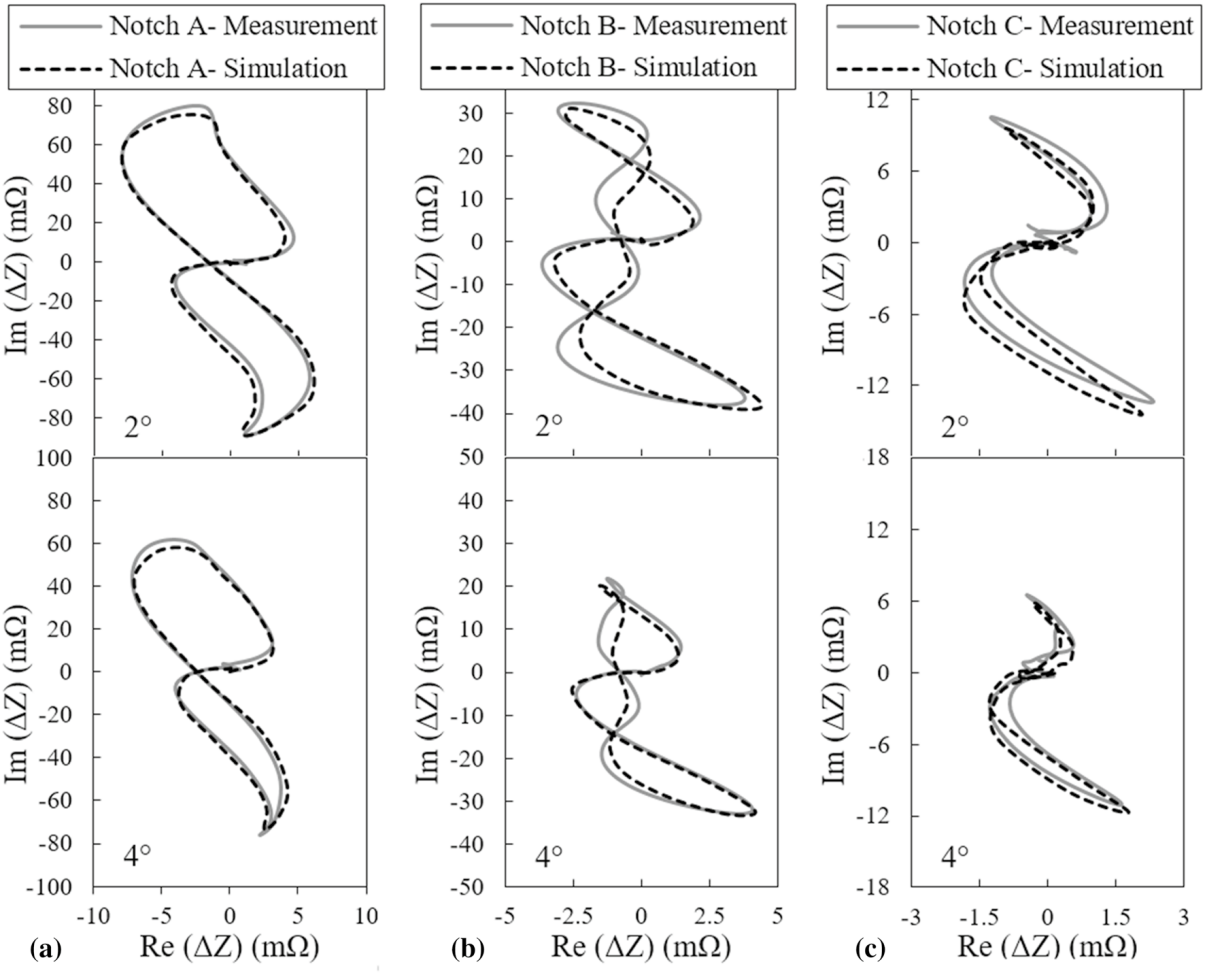
Comparison between the measured and simulated signals of **a** notch A, **b** notch B, and **c** notch C for two probe tilts of 2° and 4°

The previously presented validation strategy for lift-off variations is employed again to assess the performance of COMSOL Multiphysics® in tilt modelling. Therefore, the three calibration notches are scanned with the probe having tilt angles of 2° and 4°. Subsequently, as illustrated in Fig. 6, the simulated signals are plotted alongside the probe’s measurements for comparison purposes.

**Fig. 7:**
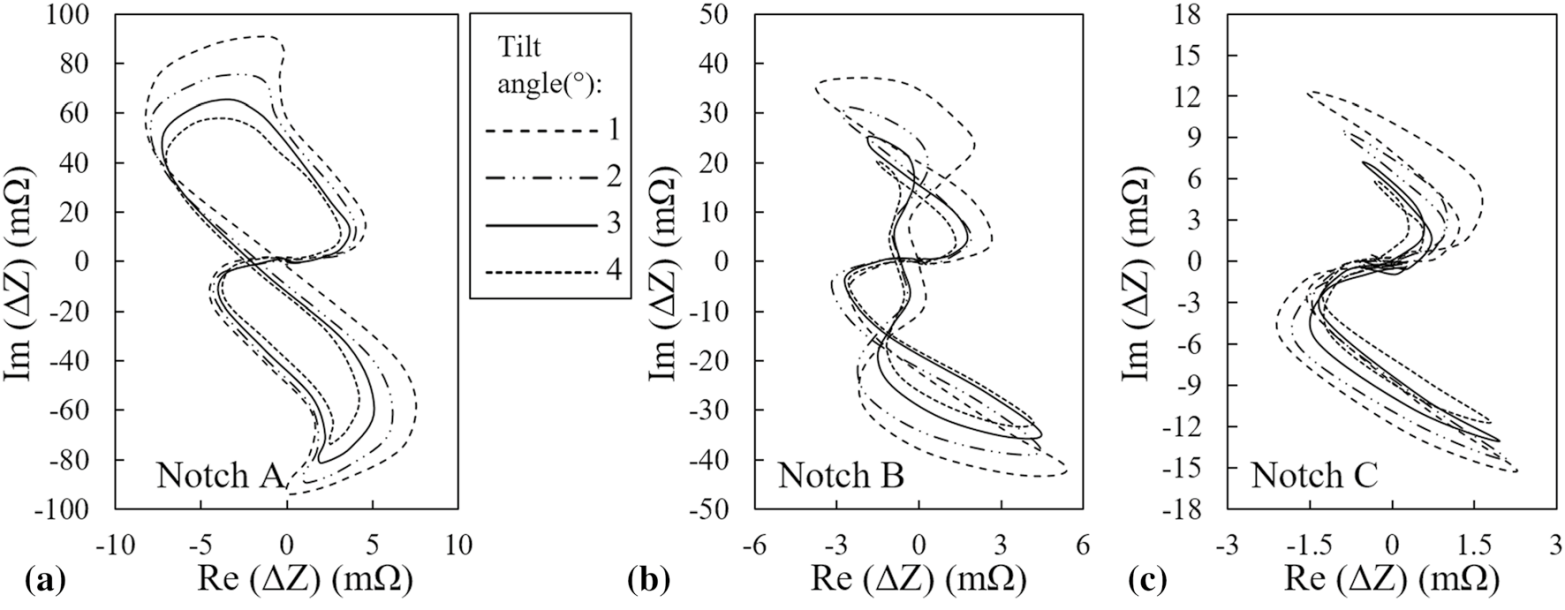
Effect of the probe’s tilt angle variations on the signal of **a** notch A, **b** notch B, and **c** notch C

The slight discrepancies between the signals presented in Fig. 6 are originated from the same sources that are listed previously with regard to the validation of the lift-off study. The influence of these estimation errors on the signal features that are used for ANFIS training is trivial and thus, the predictions are sufficiently satisfactory serving that purpose. For this reason, additional tilt angles of 1° and 3° are also simulated. The signal variations as the tilt angle varies from 1° to 4° with steps of 1° are depicted in Fig. 7.

**Fig. 8:**
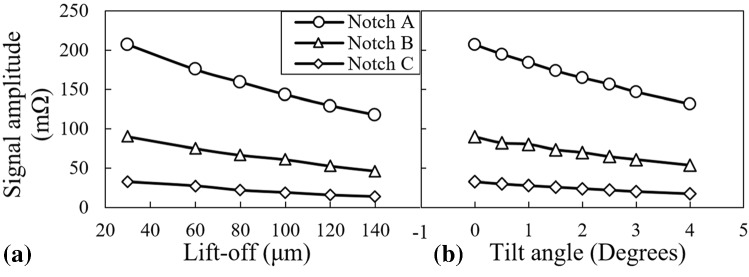
Variations of the probe’s signal amplitude as it scans three notches A, B and C versus the probe’s **a** lift-off, and **b** tilt angle

According to Figs. 5 and 7, the maximum signal amplitude of each notch decreases as either the lift-off distance or tilt angle grows whereas the signal phase changes are insignificant. Variations of the signal amplitude versus the lift-off distance and tilt angle are plotted in Fig. 8a and b, respectively. In these figures, it can be observed that the probe’s signal amplitude variations are the largest when it scans notch A as either lift-off or tilt varies. As the notch gets smaller, the variations of amplitude reduce. This phenomenon is directly connected to the notch size. In fact, notch A with a depth almost equal to 7*δ* is counted as a broad barrier to eddy current flow. Therefore, this notch perturbs a high percentage of eddy currents regardless of the density imposed by either the lift-off distance or the tilt angle. Accordingly, increasing the lift-off/tilt reduces the absolute value of the signal amplitude remarkably for this notch. As for the smallest notch C, possessing a depth equivalent to 2*δ*, the interaction with eddy currents is less as compared to the previous case of notch A. Meaning that eddy currents partially flow beneath the notch. Thus, as a result of weaker interaction between the notch and eddy currents, the signal amplitude is much smaller and hence, the variations of the amplitude caused by the lift-off/tilt changes is limited to the changes of EC density in depth of 2*δ*. So, a minor amplitude variation is observed for notch C as compared to the larger notches. However, plotting the normalized amplitude variations, as shown in Fig. 12, would prove that the amplitude of the smallest notch has higher sensitivity to these lift-off/tilt variations. Clearly, the behavior of amplitude variations for notch B falls in between the other two.

### Size Dependent Signals for ANFIS Training

**Fig. 9:**
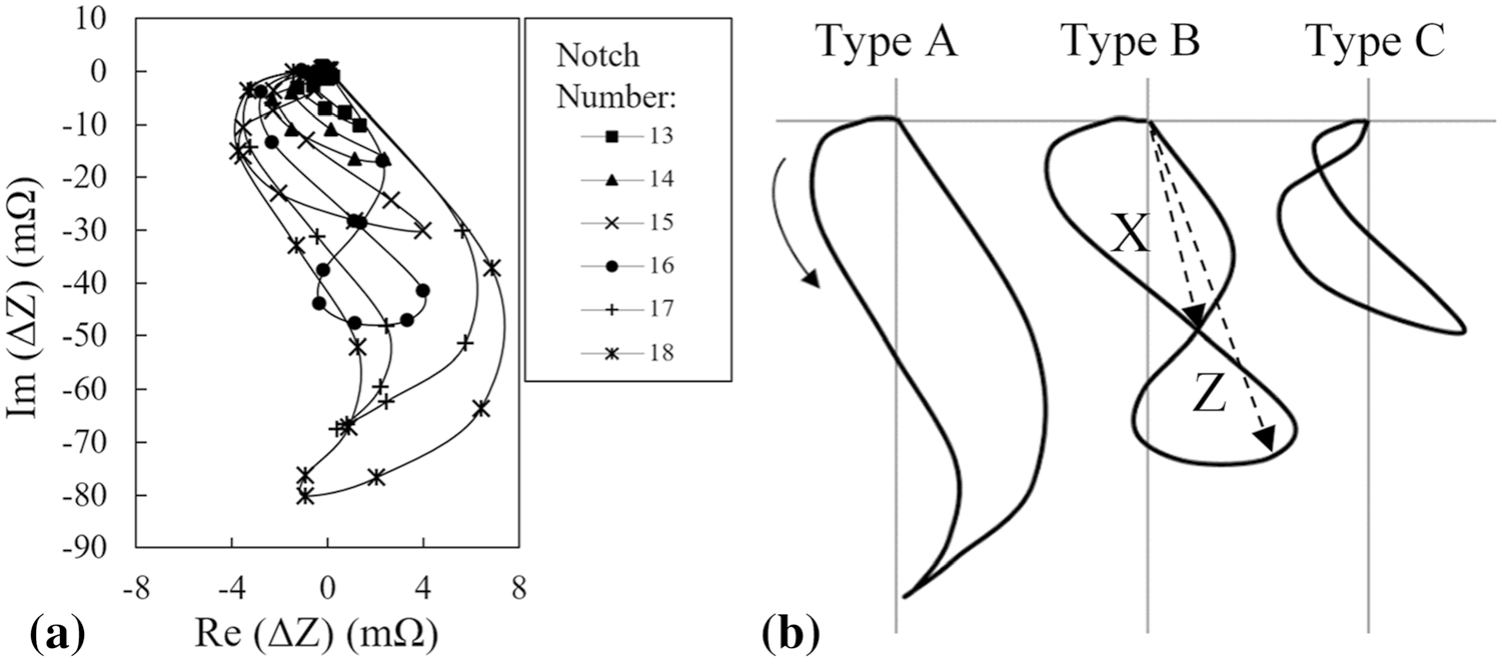
**a** Variations of the form of the probe’s signal as it scans 6 different notches with different dimensions and **b** three signal shape categories used for training ANFIS

The agreement between the signals obtained by simulations with those of measurements when the probe has its initial lift-off and does not have any tilt angle is previously verified for three calibration notches confirming the versatility of the model in predicting the signals of notches having different sizes as well as the consistency of the outcomes [16]. Accordingly, the model is used henceforth to simulate the scan of thirty notches presented in Table 2. Simulated signals of the notches numbered 13 to 18 are presented in Fig. 9a. As it can be seen, apart from the variations of the signal phase and amplitude, the signals can be classified into three distinctive shape classes which can be correlated to the ratio of the notch length to the probe’s diameter. These three types can be discriminated by the number of the loops that appears in impedance trajectory. A schematic of these three types are presented in Fig. 9b. As depicted for type A, the half-scaled trajectory made by the movement of impedance loci forms only one loop as the probe scans a notch until it reaches the notch center. Both the B and C type half-scaled signals have two loops however, for the type B signal, the magnitude of vector ***X*** connecting the impedance plane’s origin to the point at which the curve intercepts itself is larger than 20% of the vector ***Z*** magnitude (i.e. the half of the peak to peak signal amplitude). Beside other parameters, the type classification presented here is used as a training input for ANFIS in the next chapter.

## Adaptive Neuro-fuzzy Inference System

**Fig. 10:**
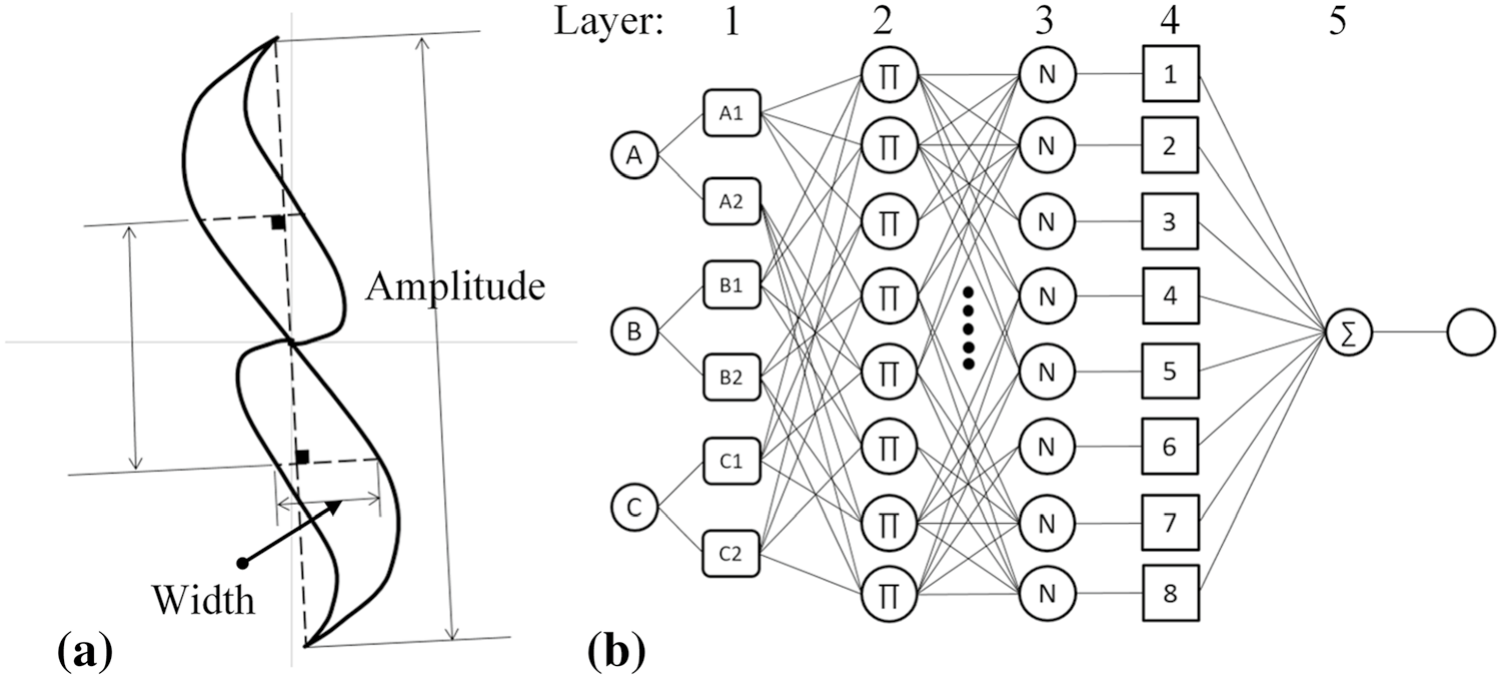
**a** Maximum signal amplitude and width shown on a simulated signal of notch A and **b** structure of a three inputs/single output ANFIS [17]

A grid type ANFIS with an initial three inputs/single output Sugeno FIS is used in training. The set of inputs/output data used for this system is listed in Table 3. In Fig. 10a, the signal features used as inputs to ANFIS are marked on one of the simulated signals. Noteworthy, on each signal, the point at which the signal amplitude becomes maximum is determined and connected by a line ***Z*** to the plane’s origin. Afterwards, the hypothetical lines perpendicular to the line ***Z*** that intercept the impedance trajectory at two points are considered and the distance between the two points on each of these hypothetical lines is measured. Accordingly, the maximum distance is taken as the signal width. These features are calculated using a MATLAB script for all thirty signals.

**Table 3:** Set of inputs and output that are used in the form of a vector to train a grid type ANFIS

Inputs			Output
Amplitude (Ω)	Maximum width (Ω)	Type (A, B and C)	Notch length (mm)

The architecture of a grid type ANFIS having three inputs and a single output is presented in Fig. 10b. The demonstrated system is comprised of five layers where in layer one fuzzification happens meaning that, the membership value of the input is determined through fuzzy sets and the membership function defined for each input. In second layer, an AND operation representing the fuzzy rule is performed at each node providing an output derived from the algebraic product of the membership values of the previous layer. In third layer, each output of layer 2 is divided by the sum of all outputs for the purpose of normalizing and in layer 4, each normalized value from previous layer is multiplied by the sum of inputs having adjustable coefficients. Last layer yields the final output by performing summation on the outputs of layer 4. As it can be seen in Table 2, the simulated signals used for training the ANFIS are mostly dependent on the notch length since none of these notches share the same length. Accordingly, the system is trained by having the notch length as output. In Table 2, it can be observed that from one column to the other, the notch depth does not change while the length varies. In spite of the fact that their depth is analogous, their signal and hence, extracted features vary since the notch lengths change. Therefore, training the system with depth as an output results in inconsistency in learning which leads to unreliable depth sizing. In other words, the table of signals prepared in this study is primarily generated for notch length estimation and it is not fit for training a depth estimation system.

Three Gaussian curve membership functions are assigned to each of the FIS inputs. In addition, the system’s output is taken constant and weight average defuzzification method is used to obtain the final output [17]. Afterwards, the designed FIS is imported to ANFIS and trained by means of the thirty inputs/output vectors in the form of the one presented in Table 3. Each of these vectors is associated to one of the notches listed in Table 2. The structure of the resulting system has 256 fuzzy rules. The error generated during the training of the system is 2%. This error corresponds to the difference between the outputs fed to ANFIS and the predictions of the trained system for the given inputs. Performance of the system is also tested by measuring the error in length estimation for the three calibration notches as well as 5 extra notches modelled in COMSOL Multiphysics®. To this end, the features for both measured and simulated signals are inserted into the trained FIS. Accordingly, Table 4 enlists both actual and estimated lengths of aforementioned test notches for which the length estimation error is also presented. The average error in length estimation of the test notches does not exceed 5%. However, this error for Tests 1, 2 and 3 is larger than 5%. The relatively larger error for these three checking data is due to the fact that the notch depths used to generate the checking data are sufficiently different from the notch depth values of the training data. Noteworthy, the notch depth population is only 20% of the population of notch length in the training data. These errors can be reduced by using more data for training, and by modifying the choice of membership functions including their number and types [17].

**Table 4:** Nominal length and the length estimated by the trained FIS for 3 calibrations and 5 model-based EDM notches

Notch name	Nominal length (mm)	Estimated length (mm)	Error (%)	Notch name	Nominal length (mm)	Estimated length (mm)	Error (%)
Notch A	2.84	2.86	0.70	Test 2	1.35	1.48	9.63
Notch B	1.62	1.68	3.70	Test 3	1.85	2.01	8.65
Notch C	0.81	0.83	2.47	Test 4	2.50	2.61	4.40
Test 1	1.20	1.29	7.50	Test 5	3.00	3.03	1.00

**Fig. 11:**
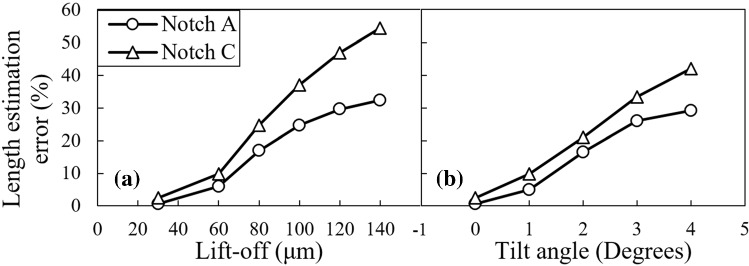
Percentage of additional error introduced into the length estimation of notch A and C because of the probe’s **a** lift-off and **b** tilt angle variations

**Fig. 12:**
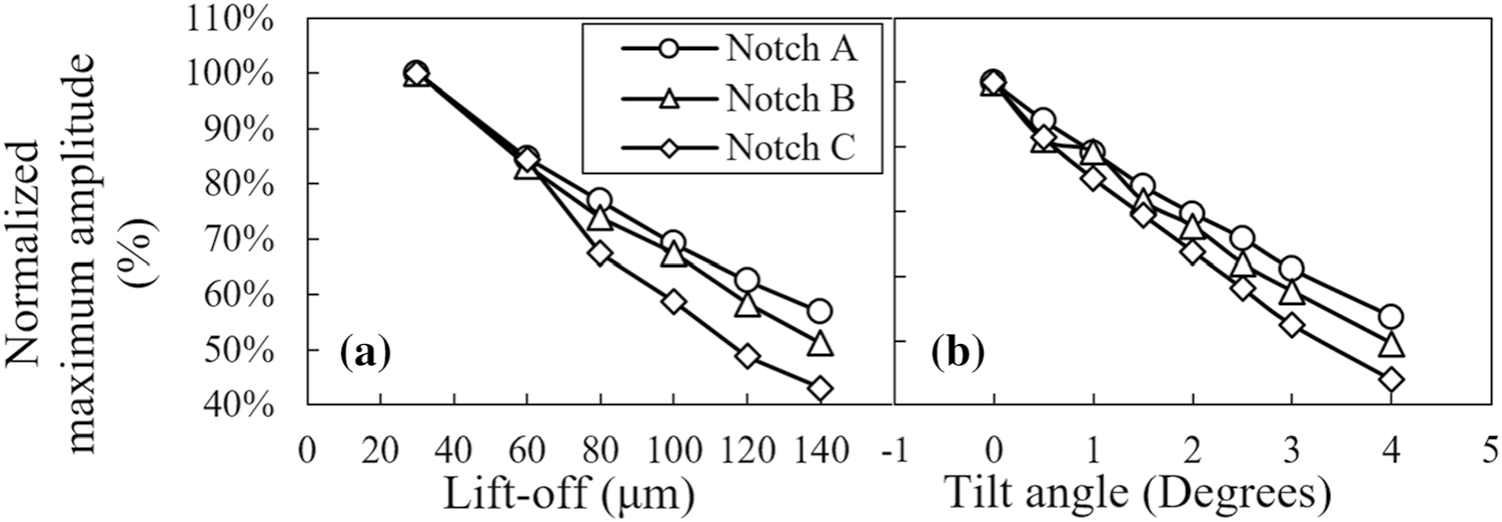
Variations of the normalized maximum amplitude of three notches A, B and C as the probe’s **a** lift-off and **b** tilt angle change

In the second phase of the study, the error introduced in length estimation caused by small variations of the probe’s tilt and lift-off are examined for the longest and the shortest calibration notches. Their signals as the probe’s lift-off and tilt change are previously depicted in Figs. 5 and 7, respectively. These signals are processed to extract their features which are subsequently fed into the trained FIS. Accordingly, the additional error in length estimation caused by tilt and lift-off variations are acquired and plotted in Fig. 11. According to this figure, the error connected to the length estimation made by FIS grows for both notches A and C as either the lift-off or the tilt angle of the probe increases. Moreover, the error associated to notch C grows with a higher rate as compared to notch A. To understand the phenomenon supporting the fact, it would be beneficial to plot the normalized signal amplitudes versus the lift-off and tilt variations for the three calibration notches, as depicted in Fig. 12a and b, respectively.

For the plots of Fig. 12, the maximum signal amplitude obtained for each notch is normalized by the maximum amplitude of the same notch at the lowest lift-off/tilt value. Looking at this figure, it is clear that the sensitivity of the signal amplitude to both lift-off and tilt increases as the notch gets smaller yielding the highest sensitivity for the smallest notch C. Since, the interaction of this notch with eddy currents is less significant as compared to the case of notch A, it has a smaller signal amplitude. Besides, the notch is shallower than the first standard penetration depth meaning that it interacts with high density current flow. Considering these two facts, it would be evident that the smallest changes in the lift-off/tilt which in turn changes the eddy current density can have a more significant impact on the normalized amplitude of the smaller notches. In Fig. 12, the rate of changes in the maximum signal amplitude of notch C, which is an input to the trained FIS, is higher than the notch A versus tilt/lift-off variations and hence, the error introduced into sizing would increase more rapidly. Furthermore, looking at each of the curves plotted in Fig. 11 individually, it can be seen that there exists a turning point on each curve dividing it to two zones. Before the turning points the sizing error grows faster while the growth rate diminishes as the point is passed. One could expect these observations concerning the error curves since according to eddy current principles, the density of induced currents changes remarkably as the probe is lifted within very low lift-off distances. On the other hand, the variations in current density becomes less sensitive to lift-off changes when the probe is more distant from the sample and the coupling is weaker.

## Conclusions

According to the results presented in the preceding sections concerning the effect of the probe’s tilt and lift-off on eddy current signal of the notch as well as the ANFIS training and testing of the trained FIS the following results can be concluded:

- Based on the validation studies carried out here, it is found that COMSOL Multiphysics® reliably predicts the behavior of eddy current signal variations as the probe’s lift-off distance and tilt angle varies. The maximum signal amplitude decreases as either the lift-off or tilt increases since the coupling between the probe and the test piece weakens. These amplitude variations are greater for the largest notch A and it becomes less significant as the notch gets smaller. However, the plot of the normalized impedance versus lift-off/tilt shows that the sensitivity of the normalized impedance to the changes of lift-off/tilt increases as the notch gets smaller.
- The size dependent table of the signals defined in this study is designed to provide ANFIS training data for length estimation. This table is comprised of thirty notches having different lengths. Obviously, the depth diversity of the notches modelled here is not sufficient to provide an efficient training for depth estimation.
- The trained FIS could estimate the length of the test notches with an average error of less than 5%, where 2% of this error is attributed to the system’s inherent training error. The length estimation error for the measured signals of the calibration notches A, B and C stays less than 2.5% proving that the system can be used effectively to perform length estimation regardless of the length to depth ratio for semi-elliptical notches in this material.
- It is observed that by increasing the probe’s lift-off/tilt the system underestimates the length of the calibration notches. Moreover, the growth rate in length estimation error as the lift-off/tilt increases is lower at the beginning, and it intensifies as the lift-off/tilt increases. This growth rate drops at higher lift-off distances and tilt angles. In addition, the rate of the variations for the length estimation error is the highest for the smallest notch C since its signal demonstrates to be the most sensitive to the lift-off/tilt changes.
